# Polypus in the External Auditory Canal

**Published:** 1885-05

**Authors:** A. W. Calhoun

**Affiliations:** Professor of Diseases of the Eye, Ear and Throat, Atlanta, Ga.


### POLYPUS IN THE EXTERNAL AUDITORY CANAL.

A CLINICAL LECTURE DELIVERED AT THE ATLANTA MEDICAL COLLEGE, BY A. W. CALHOUN, M. D., PROFESSOR OF DISEASES OF THE EYE, EAR AND THROAT, ATLANTA, GA.

Reported for the ATLANTA MEDICAL AND SURGICAL JOURNAL.

The case I now present you, gentlemen, is unlike any I have yet shown you. This patient, a stout, healthy-looking man, 23 years old, has a polypus in the right ear. It is so large that it not only fills the canal, but protrudes from the external auditory meatus, as you can plainly see.

The patient says that, when a mere child, he had a "running" ear, and that the discharge has continued from that time until the present. Only a few months ago the polypus was discovered by the patient. Since that time it has grown to its present size without being molested. The hearing in this ear is entirely lost, and will never be restored.

You notice a profuse discharge, which is very offensive. You will also notice that the slightest interference with the polypus causes slight hemorrhage.

In this case, as in the majority of cases of the kind, the polypus grows from the mucous membrane lining the cavity of the tympanum.

This polypus is the result of chronic suppurative inflammation of the middle ear. It is very unusual for one to take its growth from the walls of the external auditory canal. This is an unusually large one. Generally, they are much smaller than this. Most frequently they are found about the size of an ordinary pea, but sometimes smaller. It is very rare to find one so large as the one you now see.

So long as the polypus continues to exist, the discharge cannot be cured. The discharge must necessarily continue so long as the polypus remains, for the polypus acts as a foreign body, and keeps up a constant irritation of an already inflamed surface.

It matters not how small a polypus may be, it is almost impossible to arrest the discharge from the ear until the polypus has been removed. Many cases will get well of the discharge after the removal of the polypus without further treatment.

The very presence of such a polypus as this one, presupposes the existence of more or less chronic inflammation in the middle ear, and perforation of the drum membrane.

### TREATMENT.

The first thing to be done in the treatment of this case is to remove the polypus, which can be readily done with a pair of aural forceps, such as I now hold in my hand.

You notice we grasp the polypus with these forceps and gently twist it off. Sometimes a small portion of the pedicle is left after twisting off the polypus, as has been done in this case. When such is the case, you should always make an application of chromic acid paste to this portion and it will soon be cured.

Small polypi may be treated by this application, with the best results, without using the forceps at all. There are a number of different applications used for this purpose, but I have found that the chromic acid paste gives better satisfaction than any other.

This paste is prepared by adding one drop of water to a few crystals of the acid and stirring it with a glass rod. The paste is best applied by means of a small pledget of cotton on the end of a probe through, the ordinary ear speculum. Several applications will sometimes be necessary to effect a cure. I believe I would oe safe in saying that a few applications of this paste would destroy any aural polypus of ordinary size.

The application is quite painful in some cases. This can usually soon be relieved by syringing the ear with a little warm water.

If the offensive discharge does not cease in a few days, after disappearance of the polypus, the ordinary treatment, consisting of absolute cleanliness and astringent applications, will be advised, and before a great while our patient will be entirely well.

Remember, gentlemen, that nothing will ever cu.e such a discharge so long as any particle of the polypus remains; therefore, always be certain that you have removed every particle of it, either with the forceps or the paste, before allowing your patients to get from under your observation.

[NOTE.—This lecture was delivered in December. Three months after the operation, the patient writes: "I am entirely well of the discharge, and have no trouble whatever with my ear. My hearing has not been restored."—REPORTER.]

